# Manner of Putting up Journals for the Mails

**Published:** 1883-07

**Authors:** 


### MANNER OF PUTTING UP JOURNALS FOR THE MAILS.

---

Will our exchanges allow us to make at least one suggestion on this point?

Among the large number of journals received at this office, we do not fail to observe with pleasure, as our brethren, no doubt, have done as well, the convenience and ease with which those periodicals are opened and handled which have been sent out in a simple cover without any folding or rolling up

Why cannot all, or most of them, be put up in the same way for transmission? It is only necessary to name in this connection the following journals, in order to remind our contemporaries of the unruffled smoothness and elegant freshness with which they always make their appearance upon our tables: The Obstetrical Gazette, Cincinnati; The American Journal of Obstetrics and Diseases of Women and Children, New York; The St. Louis Courier of Medicine; The Chicago Medical Journal and Examiner; The Quarterly Epitome of American Practical Medicine and Surgery, New York; The Independent Practitioner, Buffalo, N. Y; The American Practitioner, Louisville, Ky.; The Birmingham Medical Review, England; and the Canada Medical and Surgical Journal, Montreal.

Let every journal be wrapped for the post in the same manner, and they will be both read and filed with an ease and convenience to which we are not now accustomed in the hundreds not named. The postal department is proverbial for the care and safety of its transportations.

